# Medroxyprogesterone acetate causes the alterations of endoplasmic reticulum related mRNAs and lncRNAs in endometrial cancer cells

**DOI:** 10.1186/s12920-019-0601-9

**Published:** 2019-11-12

**Authors:** Wenjiao Cao, Wuyuan Gao, Panchan Zheng, Xiao Sun, Lihua Wang

**Affiliations:** 1 Department of Obstetrics and Gynecology, the International Peace Maternity & Child Health Hospital of China Welfare Institute (IPMCH), Shanghai Jiaotong University, No.910, Hengshan Road, Xuhui District, Shanghai, 200030 China; 2 The International Peace Maternity and Child Health Hospital, School of Medicine, Shanghai Jiao Tong University, Shanghai, China; 3 Shanghai Key Laboratory of Embryo Original Diseases, Shanghai, China; 4 Shanghai Municipal Key Clinical Specialty, Shanghai, China

**Keywords:** Endometrial cancer, Medroxyprogesterone acetate, Endoplasmic reticulum stress, CHOP, Lnc-CETP-3

## Abstract

**Background:**

Progestin is effective to promote endometrial cancer (EC) cells apoptosis, however, continuous progestin administration causes low level of progestin receptor B (PRB), further resulting in progestin resistance. Here, we performed microarray analysis on Ishikawa cells (PRB+) treated with medroxyprogesterone acetate (MPA) to explore the molecular mechanism underlying the inhibitory influence of MPA on PRB+ EC cells.

**Methods:**

Microarray analysis was performed by using Ishikawa cells (PRB+) treated with MPA. Differentially expressed mRNA and long noncoding RNAs (lncRNAs) were identified. Furthermore, the functions of these mRNAs and lncRNAs were predicted by functional enrichment analysis. QRT-PCR was further performed to verify the microarray data.

**Results:**

A total of 358 differentially expressed genes and 292 lncRNAs were identified in Ishikawa cells (PRB+) treated with MPA. QRT-PCR verified these data. Functional enrichment analysis identified endoplasmic reticulum (ER) stress as the key pathway involved in the inhibitory effect of MPA on EC cells. And the ER stress apoptotic molecule CHOP and ER stress related molecule HERPUD1 were both highly expressed in Ishikawa cells (PRB+) treated with MPA. Co-expression analysis showed lnc-CETP-3 was highly correlated with CHOP and HERPUD1, suggesting it might participate in ER stress pathway-related EC cell apoptosis caused by MPA. In addition, compared with untreated cells, lnc-CETP-3, CHOP and HERPUD1 were significantly up-regulated in Ishikawa cells (PRB+) treated with MPA, whereas they have no statistical significance in KLE cells (PRB-).

**Conclusions:**

MPA may activate ER stress by progesterone-PRB pathway to up-regulate CHOP expression, which may be one of the molecular mechanisms underlying the inhibitory effect of MPA on EC cells with PRB+. Lnc-CETP-3 might be involved in this process. These findings may provide therapeutic targets for EC patients with PRB-, and resistance-related targets to increase the sensitivity of MPA on EC cells.

## Background

Endometrial carcinoma (EC) is the most common malignant tumor of the female reproductive tract resulting in 70,000 deaths worldwide annually [1]. Medroxyprogesterone acetate (MPA), a steroidal progestin, has been used as a conservative treatment for young patients with clinical stage I, grade I EC for a long time, which is desirable in patients hoping to preserve fertility [2]. The overall response rate of progestin treatment for EC ranges from 15 to 40% [3]. When treated with MAP, 10 mg daily for 12–14 days each month, 80–90% patients have regressed hyperplasia without atypia to normal endometrium [4]. However, most patients with EC develop progestin-resistance though they initially respond to progestin treatment, which further causes tumor progression [2]. A study has reported that 62% of patients with EC have an initial response to progestin, and 23% of initial responders later have developed recurrent disease [5]. In addition, > 30% of young patients have no response to progestin due to de novo or acquired progestin resistance during treatment [6]. However, the mechanism underlying progestin resistance remains largely unrevealed.

At present, many studies have reported some possible factors associated with progestin resistance. Low expression level of progestin receptor (PR) caused by continuous progestin administration is regarded as one of the reasons since it further decreases the sensitivity of cancer cells to progestin [7]. The other molecules including up-regulation of growth factors or their receptors contribute to progestin resistance. Increased level of epidermal growth factor receptor (EGFR) reduces Ishikawa EC cells sensitivity to progestin and PR expression, and abnormally activated the mitogen-activated protein kinase (MAPK) signaling pathway [8]. Overexpression of insulin-like growth factor II (IGF-II) inhibited PR expression partially mediating through activating AMP-activated protein kinase (AMPK) and inhibiting the over activated mTOR pathway [9]. In addition, activation of the PI3K/Akt pathway by progestin without PR mediation also causes progestin resistance to EC cells, and inhibiting this pathway is considered as an effective method to reverse resistance [10]. These reports suggest that the association with PR does not fully explain the mechanism of progestin resistance. Our previous studies have found RANK-RANKL (the targeting of receptor activator of nuclear factor-κB ligand) system promotes EC progression via MAPK pathway [11] and epithelial-mesenchymal transition (EMT) [12], and induces EC metastasis mediated by AKT/β-catenin/Snail pathway both in vitro and in vivo [13]. We further find that a combination of thioridazine (THIO) and MAP significantly enhances the expression levels of MPA-mediated progesterone receptor B (PRB) and dopamine receptor D2 (DRD2), and decreases the ratio of p-AKT/AKT in PI3K/AKT signal pathway, which further inhibits EC cells proliferation and promotes cell apoptosis [14].

Long noncoding RNAs (lncRNAs) encoded by a vast less explores region of the human genome, have been reported to be biomarkers and therapeutic targets for cancer, since they play a critical in promoting and maintaining tumor initiation and progression [15]. LncRNAs interact with proteins, RNA, and lipids, suggesting that they are essential mediators of cancer signaling pathways. In addition, the dysregulation of lncRNAs is associated with the stage and prognosis of many tumor types, including EC, as well as involved in resistance against chemotherapy and targeted therapy [15]. Previous studies have identified several lncRNAs correlated with EC progression. LncRNA-GAS5 promotes EC cell apoptosis by regulating the expression of miR-103 and PTEN [16]. High expression level of lncRNA BANCR in type 1 EC tissue promotes cell proliferation, migration and invasion by activating ERK/MAPK signaling pathway [17]. LncRNA HOTAIR regulates NPM1 via interacting with miR-646, and further mediates the estrogem-induced metastasis of EC cells [18]. High levels of lncRNA MALAT1 have been reported in EC, which is associated with the aberrant activation of the wnt/beta-catenin pathway leading to the interaction of wnt-effector transcription factor TCF4 and MALAT1 promoter region [19]. Furthermore, lncRNA LINC00672 has been demonstrated to increase the sensitivity of EC xenograft mice to paclitaxel [20]. Though various lncRNAs show a relationship to EC progression, to our knowledge, the association between lncRNAs and progestin resistance in EC cells has not been evaluated.

Here, to explore the molecular mechanism underlying the inhibitory influence of MPA on PRB+ EC cells, we performed microarray to analyze the mRNA and lncRNA expression profiling between Ishikawa cells (stably expressing PRB) with MPA treatment and untreated cells. And then, differentially expressed genes and lncRNAs were identified. To predict the functions of these genes and lncRNAs in EC, we further performed functional enrichment analysis. The results showed that endoplasmic reticulum stress (ER stress) might be involved in the influence of MAP on EC progression. We further used CHOP (ER stress apoptotic molecule) and HERPUD1 (ER stress related molecule) to performed co-expression analysis, and found lnc-CETP-3 was significantly increased, and closely correlated with CHOP and HERPUD1 expression. The functional enrichment analysis based on the co-expression mRNAs of lnc-CETP-3 showed that lnc-CETP-3 was involved in cell apoptosis, cell cycle, ER stress, and cancer pathway. To validate the reliability of microarray data, we further evaluated the expression levels of selected lncRNAs by qRT-PCR. CHOP, HERPUD1 and lnc-CETP-3 were significantly increased in PRB+ EC cells, whereas they have no statistical significance in PRB- EC cells.

## Methods

### Cell culture

Ishikawa cell line (ATCC® 13,347™) and KLE cell line (ATCC® CRL-1622™) were purchased from American Type Culture Collection (ATCC). Cells were cultured in Dulbecco’s modified Eagle’s medium (DMEM) F12 medium (Hyclone # SH3026101) with 10% fetal bovine serum (FBS) (Hyclone # SH30071) and 1% Penicillin/Streptomycin (Gibco # 15140) at 37 °C in a humidified atmosphere of 5% CO_2_.

### RNA isolation and qualification

In consistent with previous studies [2, 8], 10 umol/L MPA (selleck, USA) were added into treated groups, and DMSO were added into control groups. The final concentration of DMSO did not exceed 0.1%. After 48 h, all cells were washed and collected.

Total RNA was extracted using TRIzol reagent (Invitrogen life, USA). In brief, cultured cells were washed with PBS and lysed with TRIzol reagent. After homogenization, added chloroform to TRIzol reagent. Following centrifugation, the mixture separated into two layers and the aqueous phase with RNA was transferred to a new tube. RNA was precipitated by mixing with ethanol and centrifugation. After washing with ethanol the RNA was dissolved in DEPC-treated water. NanoDrop ultraviolet spectrophotometer (Thermo Fisher Scientific, Waltham, MA, USA) was used to evaluate the purity of RNA with a 260/280 ration of ~ 2.0 and a 260/230 ratio between 2.0–2.2. An average of 10 μg RNA was purified using RNeasy Mini Kit (Qiagen, Hilden, Germany). The isolated RNA was stored at − 80 °C until assayed.

### Sequencing analysis

The purified RNA was used to construct cDNA libraries by using TruSeq RNA Sample Preparation Kit (Illumina, San Diego, CA, USA) according to manufacturer’s instruction. Following first and second cDNA strands synthesis, samples were then amplified and transcribed into fluorescent cRNA. The labeled cRNAs were further purified, and reverse transcribed to 2nd-cycle cDNA. After purification and fragmentation, 2nd-cycle cDNA was then labeled. The hybridization solution mixed with labeled cDNA, hybridization buffer and other buffers was divided into the gasket slide and arranged on the lncRNA expression microarray slide. The slides were incubated in Hybridization Oven (Affymetrix) at the temperature of 45 °C for 16 h. After hybridization, the arrays were washed, fixed, and last scanned by using a scanner (Affymetrix).

### Differentially expressed mRNA and lncRNA analysis

To identify differentially expressed mRNAs and lncRNAs, the expression levels in EC cells treated with MPA were compared with their respective expression levels in untreated cells. Statistical significance was identified through the filtering of both *P* value and FDR. |Log2(Fold change)| > 1 and *P* < 0.05 were set as threshold.

### Functional enrichment analysis

To predict the functions of the differentially expressed mRNAs or lncRNAs, Gene Ontology (GO) and Kyoto Encyclopedia of Genes and Genomes (KEGG) analyses were performed [21]. GO terms included three parts, molecular function (MF), biological process (BP) and cellular component (CC). Hypergeometric distribution test was performed to calculate the significance of each term or pathway. *P* < 0.05 was set as a threshold. The Pearson correlation coefficient between each lncRNA and mRNA was evaluated. When *p* value was less than 0.05, and the absolute value of Pearson correlation coefficient was more than 0.7, the lncRNA and mRNA were correlated.

### Co-expression network analysis

To identify the interactions between mRNAs and lncRNAs, we performed co-expression network analysis. CHOP and HERPUD1 were selected as center genes, and we screened the lncRNAs co-expressed with the two genes. LncRNA-mRNA interaction analysis was performed by popular target prediction software, and network was constructed by Cytoscape software.

### Quantitative real-time PCR analysis

Total RNA was reverse transcribed to cDNA by using SuperScript™ III Reverse Transcriptase (Invitrogen, Carlsbad, CA) according to the manufacturer’s protocol. The expression levels of circRNAs were determined by ViiA 7 Real-time PCR System (Applied Biosystems, Foster City, CA). Triplicates were performed for each sample in three independent experiments. GAPDH serves as control. Primers: for lnc-CETP-3, 5′-TCAGGGTGCTGCTGTGATTA-3′ (forward), 5′-CACAGTGAGACTTACTCAAGAA-3′ (reverse); for lnc-SLC-12A3–1, 5′-CTGCCTCTTCATGATGGT-3′ (forward), 5′-CCAACTTCTTTTATTACTCCCC-3′ (reverse); for lncSAP130–1, 5′-ACAAAGTCGTTGAAATGGTAGT-3′ (forward), 5′-GTTAACAAGCAGTGGGTCTC-3′ (reverse); for LncATF3–3, 5′-CTGTCTGGACTGTGCTAAT-3′ (forward), 5′-TTGGTTCCCAAGGGACAA-3′ (reverse); for GAPDH, 5′- TCGGAGTCAACGGATTTGGT-3′ (forward), 5′- TTGCCATGGGTGGAATCATA-3′ (reverse). The relative mRNA expression was calculated using the equation: 2^-∆∆Ct^.

### Statistical analysis

Data of qRT-PCR were presented as mean ± SEM. The comparisons between two groups were performed using Student’s t test. A value of *P* < 0.05 was considered to be statistically significant.

## Results

### Identification of differentially expressed mRNAs and lncRNAs

**Fig. 1:**
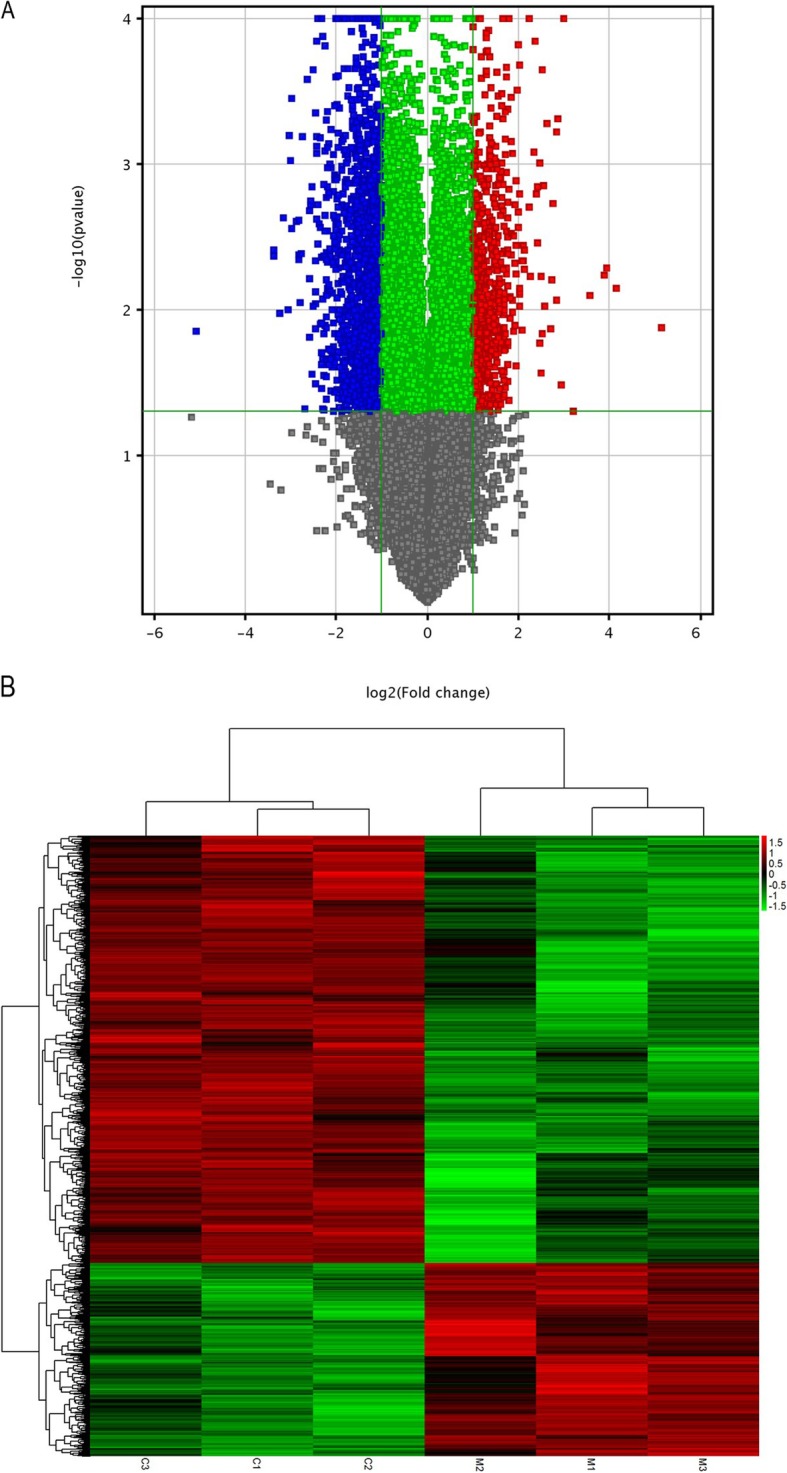
Differentially expressed mRNAs in Ishikawa cells treated with MPA compared with untreated cells. **a** The Volcano plot. Red represents significantly up-regulated mRNAs, blue represents significantly down-regulated mRNAs. **b** The heat map. Left three column represent control cell samples, right three column represent cells treated with MPA. Each raw represents each mRNA. Red represents significantly up-regulated mRNAs, green represents significantly down-regulated mRNAs

We identified the differentially expressed mRNAs and lncRNAs by comparing the expression data between Ishikawa cells treated with MPA and untreated cells. |Log2(Fold change)| > 1 and *P* < 0.05 were set as threshold. The results showed 1136 differentially expressed genes and 947 lncRNAs (Table 1). Interestingly, the number of down-regulated genes or lncRNAs was more than twice that of up-regulated genes or lncRNAs. The volcano plot showed the distribution of all the genes analyzed and highlighted the differentially expressed genes in Ishikawa cells treated with MPA (Fig. 1a). The heatmap analysis of these differentially expressed genes further demonstrated that the control samples clustered together and were separated from MPA-treated samples (Fig. 1b).

**Table 1:** The number of differentially expressed mRNA and lncRNA in Ishikawa cells treated with MPA compared with untreated cells

Differentially expressed mRNAs			Differentially expressed lncRNAs		
Up	Down	Total	Up	Down	Total
358	778	1136	292	655	947

### The functional enrichment analysis based on differentially expressed genes

**Fig. 2:**
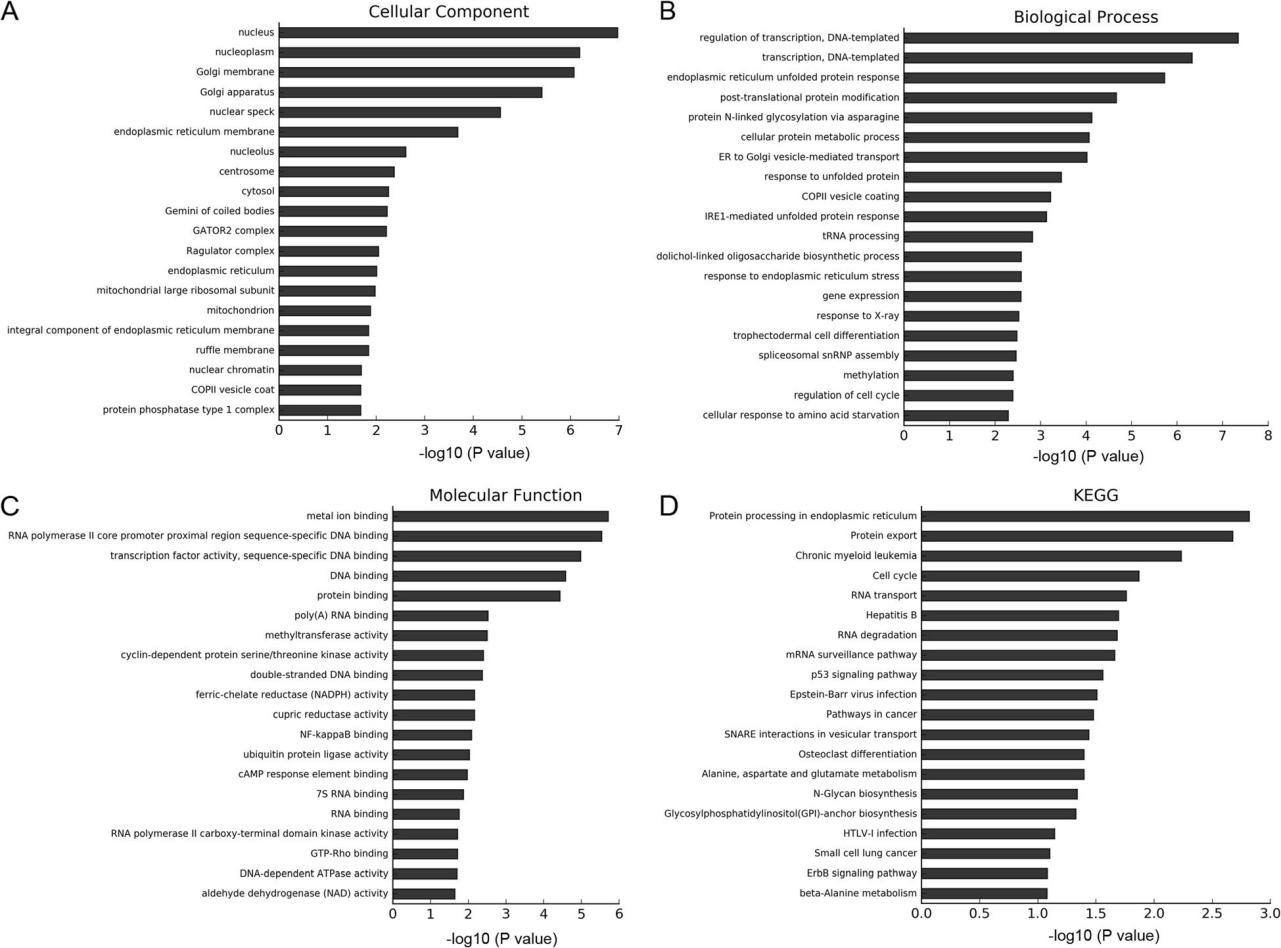
Functional enrichment analysis based on differentially expressed mRNAs. **a** GO terms in relation to cellular component. **b** GO terms in relation to biological process. **c** GO terms in relation to molecular function. **d** KEGG pathway

To evaluate the possible functions of the differentially expressed genes, we analyzed the Gene Ontology (GO) terms and Kyoto Encyclopedia of Genes and Genomes (KEGG) pathways. GO project divides into three categories, including cellular component, biological process, and molecular function. The cellular component terms showed that the differentially expressed genes were mainly located in nucleus, endoplasmic reticulum, and Golgi apparatus (Fig. 2a). The biological process terms showed they were involved in cell proliferation, metabolic process, ER stress, and unfolded protein response (Fig. 2b). The molecular function terms showed that these genes were involved in gene transcription, and protein expression (Fig. 2c). Among the top 20 significantly enriched GO terms, four terms were related to ER stress/unfolded protein response, including endoplasmic reticulum unfolded protein response, response to unfolded protein, IRE-1mediated unfolded protein response, and response to endoplasmic reticulum stress (Table 2).

**Table 2:** The biological processes related to ER stress/unfolded protein response

GO ID	term	*P* value
GO: 0030968	endoplasmic reticulum unfolded protein response	1.9E-06
GO: 0006986	response to unfolded protein	0.00035
GO: 0036498	IRE-1mediated unfolded protein response	0.00074
GO: 0034976	response to endoplasmic reticulum stress	0.00026

KEGG pathway analysis revealed that the differentially expressed genes were mainly enriched in protein processing in endoplasmic reticulum, protein export, cell cycle, and other tumor-related pathways (Fig. 2d).

### The expression levels of genes in ER stress pathway

In line with the above GO terms and KEGG pathway analysis, CCAAT/enhancer-binding protein homologous protein (CHOP), a specific marker of ER stress, was further examined and found to be highly expressed in Ishikawa cells treated with MPA. Similarly, other ER stress-related genes were also up-regulated, such as HERPUD1, ATF6 and GADD34 (Table 3).

**Table 3:** The expression levels of mRNAs related to ER stress

mRNA	Fold change	*P* value
CHOP	35.37	0.013
HERPUD1	11.97	0.008
ATF6	2.092	0.035
PPP1R15A	2.603	0.007

### The function prediction of differentially expressed lncRNAs

**Fig. 3:**
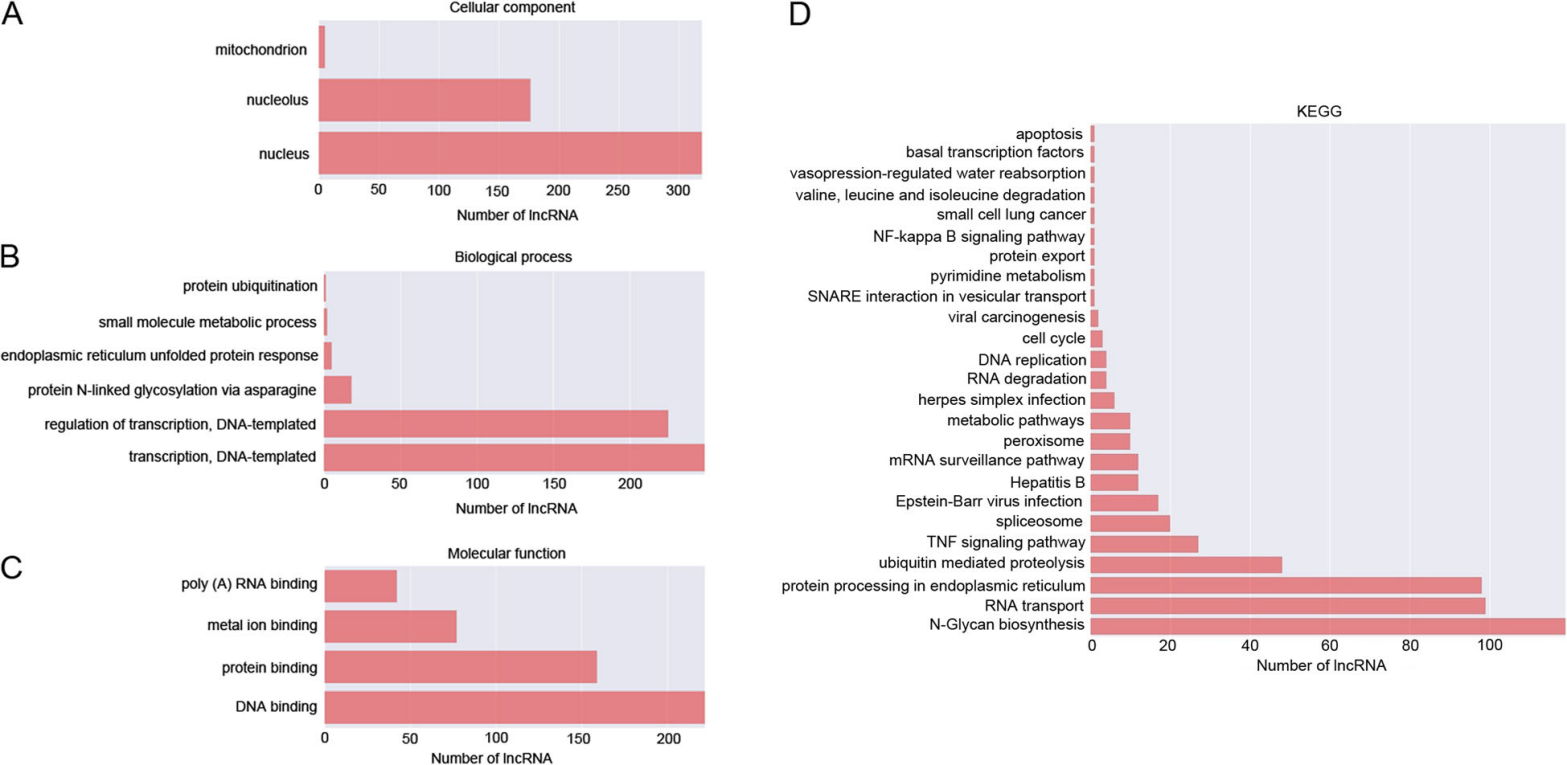
Functional enrichment analysis based on differentially expressed lncRNAs. **a** GO terms in relation to cellular component. **b** GO terms in relation to biological process. **c** GO terms in relation to molecular function. **d** KEGG pathway

To predict the function of differentially expressed lncRNAs, we selected 400 lncRNAs (the top 200 significantly up-regulated lncRNAs and the top 200 significantly down-regulated lncRNAs), and evaluated the correlation between each lncRNAs and the differentially expressed genes. Each lncRNA has a group of co-expressed genes. Thus, we tried to perform GO and KEGG analysis based on the group of genes to investigate the pathways this lncRNA might be involved in. Among the top 500 significant GO terms, we found that in Ishikawa cells treated with MPA most of the lncRNAs were associated with cell nucleus and mitochondrion in cellular component terms (Fig. 3a); in biological process terms most of the lncRNAs were involved in DNA transcription, protein ubiquitination, endoplasmic reticulum unfolded protein response, small molecule metabolic process, and protein ubiquitination (Fig. 3b); in molecular function most of the lncRNAs were involved in DNA binding, protein binding, RNA binding, and metal ion binding (Fig. 3c). KEGG pathway analysis showed that most of the lncRNAs were involved in N-Glycan biosynthesis, RNA transport, protein processing in endoplasmic reticulum, metabolic pathways, cell cycle, and apoptosis (Fig. 3d).

### Lnc-CETP-3 was screened as key lncRNA

**Fig. 4:**
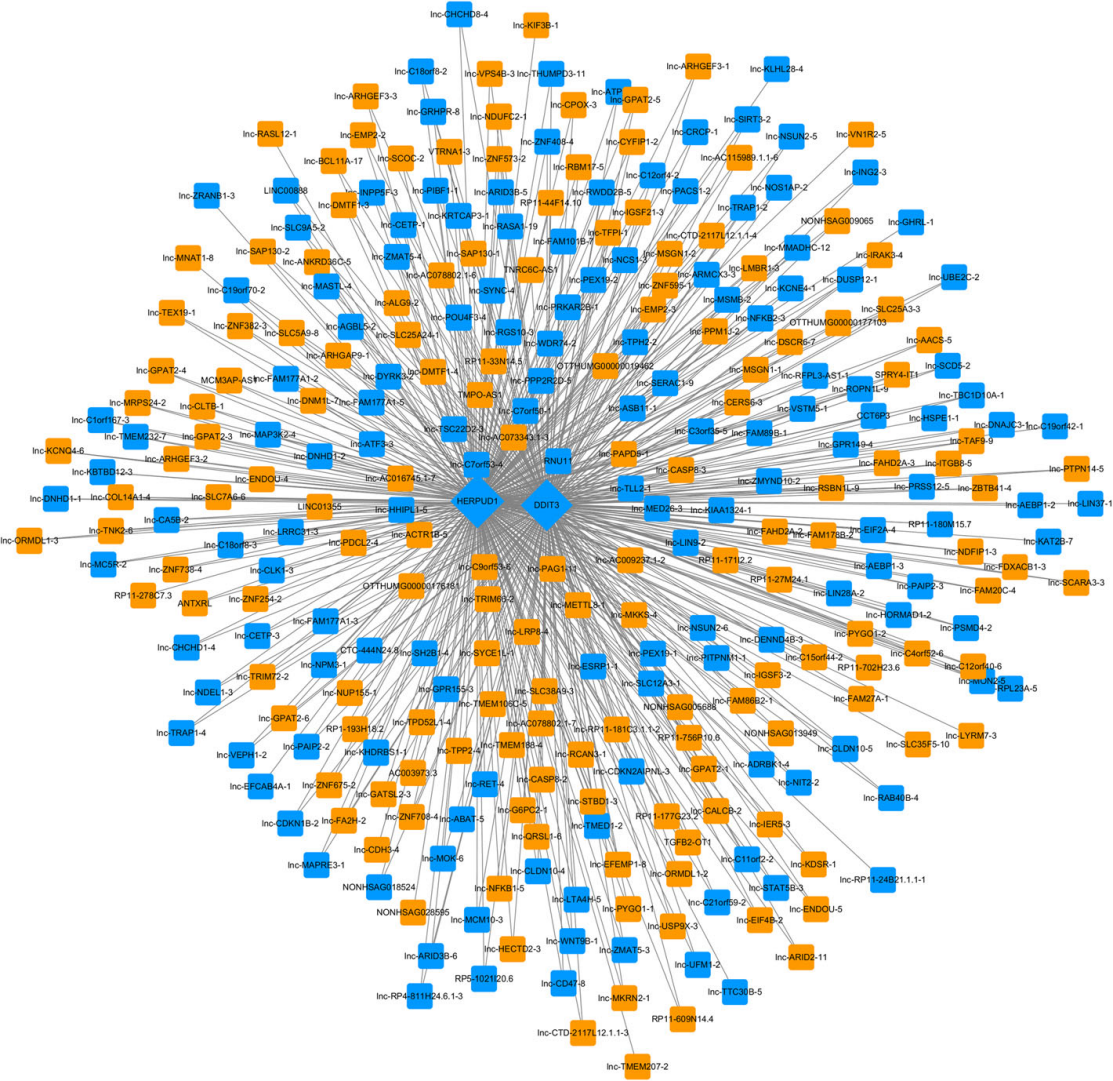
Co-expression network of CHOP, HERPUD1 and lncRNAs

To identify key lncRNAs involved in the influence of MPA on EC progression, co-expression analysis was performed. CHOP and HERPUD1 were significantly up-regulated, thus, we regarded them as hub genes to construct the co-expression network. A total of 277 lncRNAs were interacted with CHOP, and 295 lncRNAs were interacted with HERPUD1 (Fig. 4). Among them, 277 lncRNAs were interacted with both CHOP and HERPUD1.

**Fig. 5:**
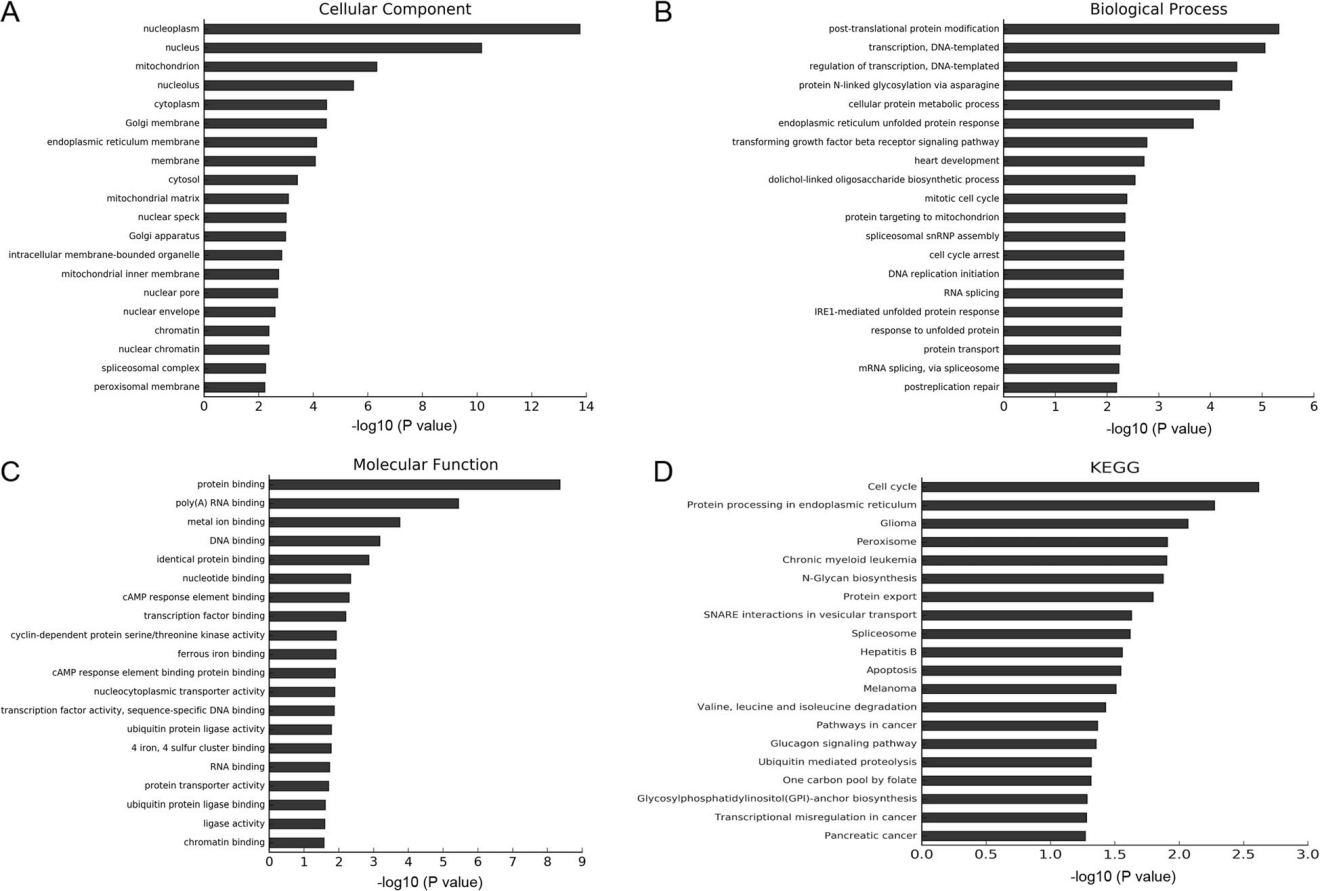
Functional enrichment analysis based on mRNAs co-expressed with lnc-CETP-3. **a** GO terms in relation to cellular component. **b** GO terms in relation to biological process. **c** GO terms in relation to molecular function. **d** KEGG pathway

Among the 277 lncRNAs, lnc-CETP-3 was highly expressed with the fold change of 15.26, and closely correlated with CHOP and HERPUD1 expression with the correlation coefficients of 0.981 and 0.999, respectively. To predict the functions of lnc-CETP-3, we analyzed GO term and KEGG pathways based on the genes co-expressed with lnc-CETP-3. GO terms analysis showed that under cellular component, terms related to cell nucleus (Fig. 5a). Under biological process, terms mainly related to protein modification, cellular protein metabolic process, protein N-linked glycosylation via asparagine, and endoplasmic reticulum unfolded protein response (Fig. 5b). Under molecular function, terms related to protein binding, poly (A) RNA binding, and cAMP response element binding (Fig. 5c). KEGG pathways analysis showed that lnc-CETP-3 co-expressed genes were mainly involved in cell cycle, protein processing in endoplasmic reticulum, apoptosis, and other tumor-related pathways (Fig. 5d). These findings suggested that lnc-CETP-3 was linked to ER stress/unfolded protein response, and tumor-related pathways.

### QRT-PCR validation

**Fig. 6:**
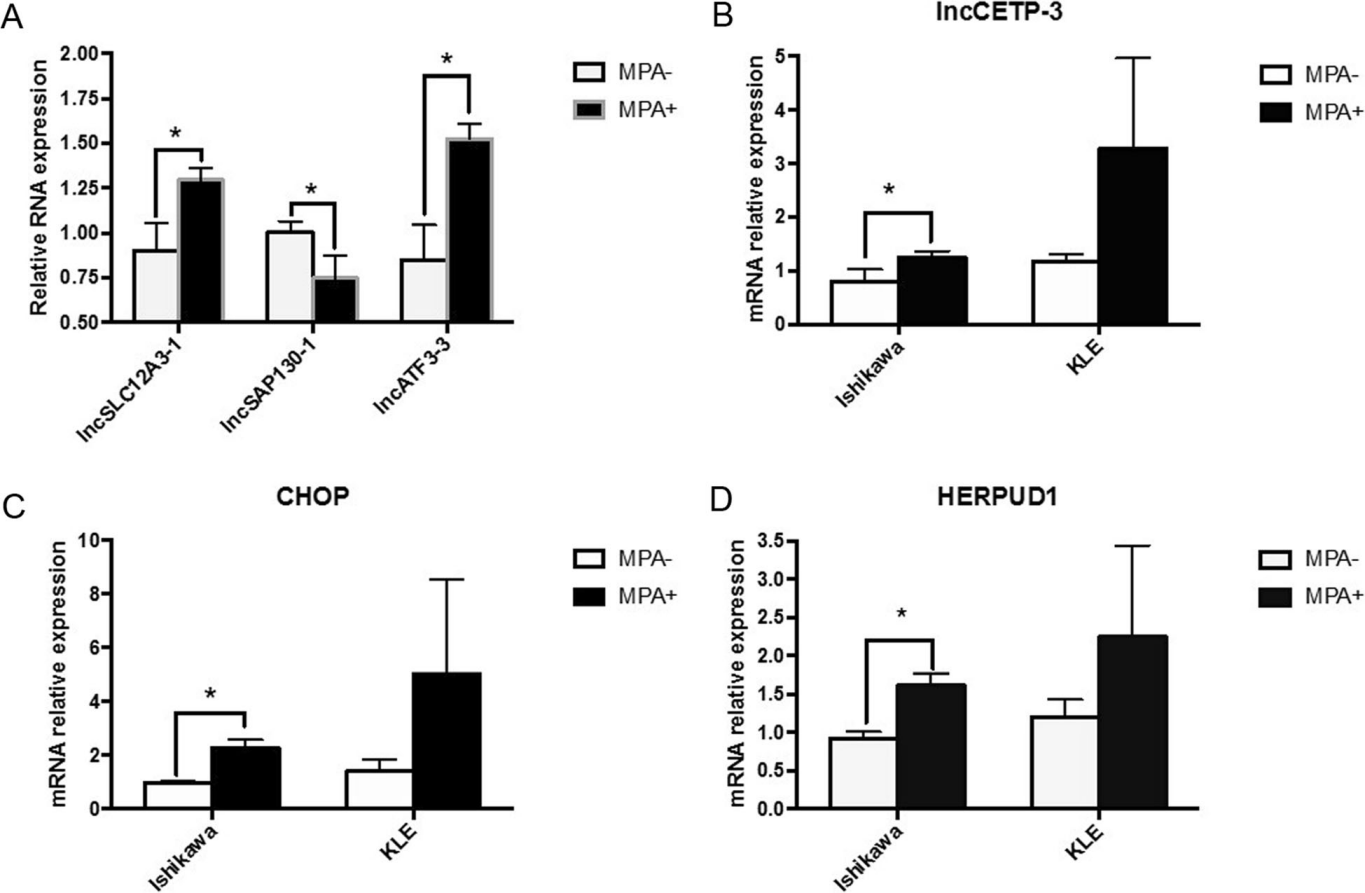
Real-time RT-PCR analysis. **a** Lnc-SLC-12A3–1 and lncATF3–3 were highly expressed in cells treated with MPA compared with untreated cells, whereas lncSAP130–1 was decreased, which were consistent with microarray data. **b** The expression of ER stress-related lncRNA lnc-CETP-3 in Ishikawa cells (PRB+) and KLE cells (PRB-). **c** The expression of ER stress-related gene CHOP in Ishikawa cells (PRB+) and KLE cells (PRB-). **d** The expression of ER stress-related gene HERPUD1 in Ishikawa cells (PRB+) and KLE cells

To verify the credibility of microarray data, we selected three differentially expressed lncRNAs, and detected their expression levels in Ishikawa cells treated with MPA by qRT-PCR. Lnc-SLC-12A3–1 and lncATF3–3 were all highly expressed in cells treated with MPA, and lncSAP130–1 was decreased, which were consistent with our microarray analysis results (Fig. 6a).

### The expression of ER stress-related genes (CHOP and HERPUD1) and lncRNA (lnc-CETP-3) in Ishikawa cells (PRB+) and KLE cells (PRB-)

Our microarray data detected the changes of mRNA and lncRNA expression profiling of Ishikawa cells stably expressing progesterone receptor B (PRB) (PRB+). To confirm that MPA induced the above expression changes by PRB, we evaluated the expression of ER stress-related genes and lncRNA, including CHOP, HERPUD1 and lnc-CETP-3, in KLE cells poorly expressing PRB (PRB-). After treated with MPA, the mRNA levels of CHOP, HERPUD1 and lnc-CETP-3 were all significantly increased in Ishikawa cells. However, their expression levels have no statistical significance in KLE cells treated with MPA compared with untreated KLE cells (Fig. 6b-d). These findings suggested that MPA might induced the mRNA and lncRNA expression changes by PRB.

## Discussion

Patients with positive-PR EC are correlated strongly with successful endocrine treatment and survival, whereas negative-PR EC are linked to progestin resistance and EC progression, since PR expression, especially PRB, is essential to progestin action [8]. Previous study has reported that PRB plays a key role in the inhibitory effects of progestin on cell growth and invasiveness, and a low PRB expression level generally represents a poor prognosis for EC patients with MPA resistance [22]. This study aimed at exploring the molecular mechanism underlying the influence of MPA on EC cells with PRB+, which may provide therapeutic targets for EC patients with PRB-, and resistance-related targets to increase the sensitivity of MPA on EC cells.

In this study, we sought to identify the differentially expressed genes and lncRNAs in PRB-positive Ishikawa cells treated with MPA. Co-expression analysis as well as GO terms and KEGG pathways analyses were all revealed that MPA activated the endoplasmic reticulum unfolded protein response in Ishikawa cells. Endoplasmic reticulum (ER), an extensive intracellular organelle, provides a site for protein modification and folding as well as intracellular calcium (Ca2+) store [23]. ER stress is characterized by accumulation of abnormal proteins, such as unfolding or misfolding proteins in the ER and overloading of Ca2+ into the mitochondria, which result from DNA damage, hypoxia, oxidative stress, and other types of physiological and pathological stimuli [24]. At the beginning of ER stress, cells activate the unfolded protein response (UPR) which is mainly regulated by three ER-located sensors, including PKR-like ER kinase (PERK), inositol-requiring enzyme 1 (IRE1) and activating transcription factor 6 (ATF6). Theses sensors initiate translational attenuation to inhibit further accumulation of misfolded proteins and to increase folding machinery, and facilitate ER-associated degradation to eliminate misfolded proteins in the ER [25]. If UPR is insufficient to alleviate the stress, it then leads to cell death [24]. Previous study has demonstrated that ER stress reduces chemotherapy resistance by down-regulating the PI3K/Akt/mTOR signaling pathway in mutant p53 lung cancer cells [26]. In this study, we found that ER stress-related genes, including CHOP, HERPUD1, ATF6 and GADD34, were highly expressed induced by MPA in EC cells. Among them, CHOP was significantly increased with the fold change of 35.37. CHOP, the best-characterized factor in the transition from ER stress to apoptosis, is expressed at low levels under physiological conditions, whereas is dramatically increased under severe and prolonged ER stress [23, 27]. It has been reported that ER stress by realgar quantum dots induces increased expression of CHOP, and further leads to EC cell apoptosis and necrosis [28]. Additionally, previous study has demonstrated that progesterone-induced ER stress may enhance EC apoptosis through overexpression of CHOP [25]. Our microarray data showed that HERPUD1 is also significantly increased with the fold change of 11.94. HERPUD1 (homocysteine-inducible, endoplasmic reticulum stress-inducible, ubiquitin-like domain member 1), an ER-resident membrane protein, facilitate ER-associated degradation, and its expression is strongly up-regulated by the unfolded protein response and cellular stress [29, 30]. We further constructed a co-expressed network based on the differentially expressed lncRNAs interacted with CHOP and HERPUD1. A total of 277 lncRNAs were interacted with both of the genes, among which lnc-CETP-3 was highly expressed with the fold change of 15.26, and closely correlated with the expression of CHOP and HERPUD1. Functional prediction analysis showed that lnc-CETP-3 was associated with endoplasmic reticulum unfolded protein response, apoptosis, cell cycle, and tumor-related pathways. These findings suggest that ER stress and lnc-CETP-3 are likely to be involved in the influence of MPA on Ishikawa cells (PRB+).

To further detect the influence of PRB on the expression of lnc-CETP-3, CHOP and HERPUD1 in EC cells treated with MPA, we selected Ishikawa cells (PRB+) and KLE cells (PRB-). QRT-PCR results showed that lnc-CETP-3, CHOP and HERPUD1 were all significantly up-regulated in Ishikawa cells treated with MPA compared with untreated Ishikawa cells, whereas their expression levels have no statistical significance in KLE cells treated with MPA compared with untreated KLE cells. These findings reveal that MPA activates ER stress and increased the expression of lnc-CETP-3 by PRB, and this may be one of the molecular mechanisms underlying the inhibitory effect of MPA on EC cells. In other word, ER stress and lnc-CETP-3 may be as therapeutic targets for patients with PRB- EC or even MPA resistance patients.

There are still many deficiencies in this study. We just predicted that MPA activated ER stress and increased the expression of CHOP to cause EC cells apoptosis, and lnc-CETP-3 might be involved in ER stress regulation. However, this prediction has not been further verified. Thus, we prepare to explore the roles of CHOP and lnc-CETP-3 in the influence of MPA on EC cells, and to confirm whether MPA-PRB-lnc-CETP-3/ERS-CHOP apoptotic pathways exist in subsequent studies. In addition, the association between CHOP, lnc-CETP-3 and PRB should be further verified in animal and human studies.

## Conclusions

In this study, we concluded that MPA activated ER stress by progesterone-PRB pathway to up-regulate CHOP expression, which may be one of the molecular mechanisms underlying the inhibitory effect of MPA on EC cells with PRB+. Lnc-CETP-3 might be involved in this process. These findings may provide therapeutic targets for EC patients with PRB-, and resistance-related targets to increase the sensitivity of MPA on EC cells.

## Data Availability

The datasets used and/or analysed during the current study are available from the corresponding author on reasonable request.

## Abbreviations

AMPK: Activating AMP-activated protein kinase
ATCC: American Type Culture Collection
ATF6: Activating transcription factor 6
BP: Biological process
CC: Cellular component
CHOP: CCAAT/enhancer-binding protein homologous protein
DMEM: Dulbecco’s modified Eagle’s medium
DRD2: Dopamine receptor D2
EC: Endometrial cancer
EGFR: Epidermal growth factor receptor
EMT: Epithelial-mesenchymal transition
ER: Endoplasmic reticulum
FBS: Fetal bovine serum
GO: Gene Ontology
HERPUD1: Homocysteine-inducible, endoplasmic reticulum stress-inducible, ubiquitin-like domain member 1
IGF-II: Insulin-like growth factor II
IRE1: Inositol-requiring enzyme 1
KEGG: Kyoto Encyclopedia of Genes and Genomes
lncRNAs: Long noncoding RNAs
MAPK: Mitogen-activated protein kinase
MF: Molecular function
MPA: Medroxyprogesterone acetate
PERK: PKR-like ER kinase
PR: Progestin receptor
PRB: Progestin receptor B
